# Predictors of prolonged hospitalization among geriatric trauma patients using the modified 5-Item Frailty index in a Middle Eastern trauma center: an 11-year retrospective study

**DOI:** 10.1007/s00068-024-02742-1

**Published:** 2025-01-24

**Authors:** Husham Abdelrahman, Ayman El-Menyar, Rafael Consunji, Naushad Ahmad Khan, Mohammad Asim, Fouad Mustafa, Adam Shunni, AbuBaker Al-Aieb, Hassan Al-Thani, Sandro Rizoli

**Affiliations:** 1 Department of Surgery, Trauma Surgery, Hamad Medical Corporation, Doha, Qatar; 2 Department of Surgery, Clinical Research, Trauma & Vascular Surgery, Hamad Medical Corporation, Doha, Qatar; 3 Department of Clinical Medicine, Weill Cornell Medical College, Doha, Qatar; 4 Department of Surgery, Trauma Surgery, Injury Prevention, Hamad Medical Corporation, Doha, Qatar

**Keywords:** Geriatric, Elderly, Trauma, Middle East, Hospitalization, Frailty index

## Abstract

**Background:**

Using a validated tool, we explored the prevalence, risk factors, and predictors of longer hospitalization among hospitalized geriatric patients.

**Methods:**

Retrospective and comparative analyses of age groups (55–64 vs. ≥ 65 years), gender, survival status, and frailty index categories were performed. The Modified 5-Item Frailty Index was assessed, and multivariable logistic regression analysis was performed to predict prolonged hospitalization (> 7 days).

**Results:**

There were 17,600 trauma hospitalizations with a mean age of 32 ± 15 years between 2010 and 2021; of them, 9.2% were geriatrics at ≥ 55–64 years (*n* = 935) and ≥ 65 years (*n* = 691). The female/male ratio was 17.5%/82.5%, and the mean injury severity score was 13 ± 9. The injury rate for age ≥ 65 was 24 per 10,000 compared to 10 per 10,000 in the younger group age (**≥** 55–64). 35% of injuries occurred at home due to falls. Overall mortality was 8%, with a higher rate among males than females (9% vs. 4%). The deceased were three years older at the time of death compared to the survivors. Higher frailty grades were associated with home-related falls and head injuries. Patients 65 years or older were likely to have higher frailty scores, as indicated by higher percentages in the mFI-5. Among the older group, 25% were moderately frail, and 18% severely frail. In the younger group, 50% were frail. Higher frailty scores correlated with increased acute kidney injury, pneumonia, urinary tract infections, and longer hospital stays. Severe frailty significantly predicted longer hospitalization (odds ratio 1.83, *p* = 0.007).

**Conclusion:**

One out of eleven trauma admissions was aged > 55. Head injury and bleeding were the leading causes of mortality in the study cohort. There was a significant decrease in the trend of geriatric trauma over the years. The modified FI-5 performs well as a predictive tool of prolonged hospitalizaion in trauma patients with different age groups.

## Introduction

Trauma is the fifth leading cause of death in the geriatric population, patients 65 years and older, and they constitute around a quarter of all trauma admissions in the Western world [1, 2]. This population is particularly unique and vulnerable due to degenerative, anatomic, and physiological changes, necessitating special attention from specialized geriatric trauma care providers [3, 4]. Geriatric patients have a higher potential for adverse outcomes after injury, even after low- impact trauma. Age-related physiological and anatomical changes, along with chronic diseases, comorbidities, and polypharmacy, can worsen clinical outcomes by reducing injury tolerance and increasing frailty. This limited physiological reserve significantly impacts the post-injury course and management in older patients [5–7]. Therefore, frailty assessment is essential to predict outcomes and inform management and decision-making [6].

As life expectancy increases, trauma centers will see more injured older adults. In the United States, the prediction is that 1 out of 5 people will be over 65 years old by 2030 [8]. While there is no consensus on the definition of the cutoff age for geriatric trauma patients, different sources widely vary and use a cutoff between 55 and 80 years [9]. The Geriatric Trauma Committee of the American Association for the Surgery of Trauma (AAST), the American College of Surgeons (ACS), and the Eastern Association for the Surgery of Trauma (EAST) Geriatric Trauma Triage Guidelines use a cutoff of ≥ 65 years to define the geriatric trauma population. However, this cutoff remains arbitrary and conventional [9–12]. However, some have reported that morbidity and mortality increased in trauma patients of ≥ 55 years [4, 10, 11, 13].

Geriatric patients are inherently different from younger adult patients in terms of socio-demographics and epidemiology. The literature describes the leading mechanisms of geriatric injury as falls followed by motor vehicle trauma and burns [4, 13]. Geriatric trauma is challenging in both its prevention and management. The associated aging process decreases physiologic reserve, and frailty negatively impacts hospital outcomes and functional recovery [14]. Nevertheless, aggressive management by multi-disciplinary teams has allowed geriatric trauma patients to gain a better quality of life as well as improved clinical outcomes [15, 16].

Most of the studies on geriatrics are from the Western world; however, the Eastern Mediterranean region lacks studies that focus on epidemiology, frailty, and management of geriatric trauma. The Arabian Gulf countries with petroleum-based economies and majority expatriate male worker populations, like Qatar, present a unique opportunity to study geriatric trauma before it emerges as a leading cause of mortality and morbidity in this population. We aim to analyze moderate to severe geriatric trauma in Qatar over 11 years in terms of incidence, risk factors, and clinical outcomes and to assess differences in characteristics between age groups in addition to the temporal trends of injury. This analysis will better define the problem of geriatric trauma in Qatar, inform public awareness programs and policies, and help formulate locally focused prevention programs.

## Methods

### Study design and population

The primary sample for this study was collected from consecutive data for all geriatric [defined as age ≥ 55 years] patients who sustained injuries and were admitted to Hamad Trauma Center [HTC]. Data were retrieved from May 1, 2010, through April 30, 2021(a total of 11 years), from the Qatar National Trauma Registry of the HTC, the only Level 1 trauma referral center in the country. HTC serves the entire country, receiving more than 95% of the county’s trauma patients, especially those with moderate and severe injuries, as well as some of the milder trauma. The trauma registry is compliant with both the National Trauma Data Bank [NTDB] and the Trauma Quality Improvement Program [TQIP] of the American College of Surgeons-Committee on Trauma [17]. It prospectively collects data on all trauma activations with different mechanisms of injury (blunt and penetrating), according to the International Classification of Diseases, Ninth Revision (ICD-9 and ICD-10) codes between 800 and 959.9.

#### Inclusion and exclusion criteria

We elected the lower limit of 55 years to define our population based on prior data that suggest this cutoff for geriatric subjects (older age as those who are 55 and above) [9, 12]. All trauma patients of age ≥ 55 with blunt or penetrating injuries admitted during the study duration were included. A study-specific data collection sheet was used to organize the extracted data from the trauma registry. Exclusion criteria included pediatric patients, patients without Frailty records, and those who were brought dead with lacking relevant data.

#### Study variables

The variables included demographic data, site of injuries such as workplace, home, or street, mechanism of injuries (MOI); blunt trauma such as road traffic injuries (RTI), fall from height and fall of heavy objects, penetrating trauma (stabs, bullets, impalement, and machine-related injuries), protective devices like seatbelt in case of MVC, helmet in bike-related injuries, and airbag deployment, place of injury, risk factors (previous fall, living alone, walking aid, depression, cognitive deficit, and use of polypharmacy), injury scores (Injury severity score (ISS), Abbreviated injury score (AIS) and Glasgow coma scale (GCS)), body sites of injuries, comorbidities, and frailty assessment using the modified frailty index five (mFI-5). The mFI-5 is an easy, fast, yet effective predictor for mortality and complications in geriatric population and can be calculated upon admission [18–20]. Other variables included shock index (SI) calculated on admission (heart rate/systolic blood pressure) [21], mode of transportation, interventions, complications, and outcomes. Injury rate: total annual admission/total geriatric population/10,000. To estimate the yearly injury rate, the total number of geriatric trauma admissions was divided by the year’s total mid-year geriatric population within specific age categories. The resulting number was then divided by 10,000 to yield the injury rate for that particular year.

#### Frailty index calculation

We retrospectively calculated the mFI-5 score using five comorbidities: diabetes mellitus (DM), hypertension (HTN), congestive heart failure (CHF), chronic obstructive pulmonary disease (COPD), and functionally dependent health status. The functionally dependent health status refers to patients who, due to their general health, are unable to complete activities of daily living independently. The total score was derived by summing the number of comorbidities present and dividing them by five. A score of 0 indicates the patient is not frail, 1 indicates mild frailty, 2 indicates moderate frailty and a score of 3 or higher indicates severe frailty [22].

#### Trauma management

The ACS Trauma triage criteria are followed by both the national pre-hospital ambulance service and the Hamad Trauma Center [HTC] (American College of Surgeons Committee On Trauma (COT). Resources for optimal care of the injured patient, 2014) [23, 24], and it includes the elderly as particular criteria for trauma activation and transfers to this trauma center, which is the sole trauma center in Qatar. All severely injured patients triaged and transferred to the HTC are initially assessed and stabilized at the trauma resuscitation unit (TRU). The ATLS guidelines are strictly followed along with other established clinical protocols. TRU has type O blood available and access to hybrid (CT scan + angio) suites. The Trauma Section oversees the entire care of the injured patients, from TRU to the trauma ICU, step-down ICU, and the trauma ward. HTC offers continuous hospital care until discharge home or to rehabilitation. Emergency care is provided at no cost to the entire population of Qatar.

#### Outcome measures

Complications across age and gender, frailty category, hospital stay, and mortality.

### Statistical analysis

Descriptive data were presented as numbers and proportions, a mean with standard deviation (SD), and median with range whenever appropriate. Patients were categorized based on age (younger 55–64 vs. older ≥ 65 years) and gender (male vs. female), and outcome (survivors vs. deceased), frailty using modified frailty index 5 (mFI-5). Pearson chi-square (χ2) test was used to compare categorical variables between two groups. The student’s t-test, analysis of variance (ANOVA), or Kruskal-Wallis’s test was applied as appropriate for continuous data. Univariate analysis was conducted to identify statistically significant variables, and those deemed clinically significant were included in multivariable logistic regression analyses. Multivariable analyses were then performed, adjusting for patient factors (age, gender, frailty score, injury severity score, shock index, renal impairment, GCS score, blood transfusion, and pneumonia to identify predictors of hospital length of stay more than a week. Data were expressed as odds ratio (OR) and 95% confidence intervals (CI). A two-tailed P value of < 0.05 was considered statistically significant. Data analysis was performed using the Statistical Package for Social Sciences (SPSS) version 28 (SPSS Inc. USA).

## Results

**Fig. 1:**
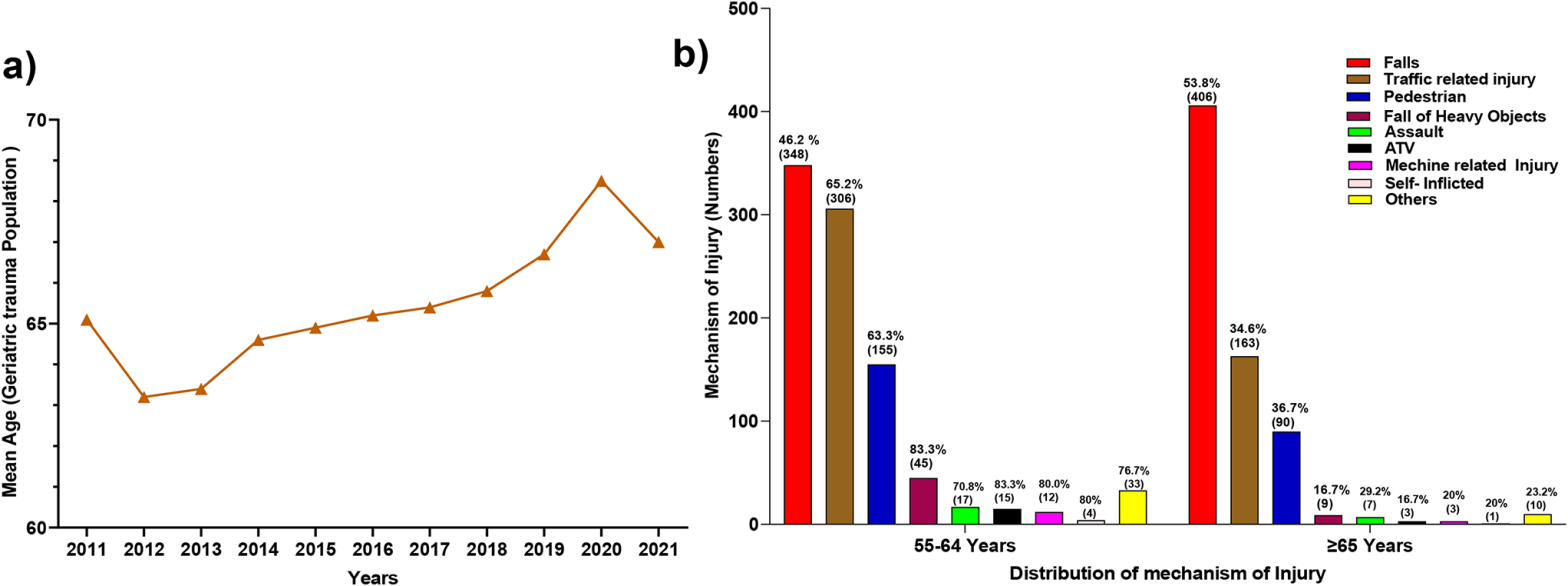
(**a**) The age trend of Geriatric trauma patients over the years. (**b**) The mechanism of injury of the trauma patients stratified by age category

Over the 11-year study period, 17,600 patients were admitted to HTC, of which 1,626 (9.2%) met inclusion criteria: 935 (57.5%) patients (aged 55–64 years) and 691 (42.5%) (aged ≥ 65 years). Males dominated with a male-to-female ratio of 4:1. Nationals (Qataris) comprised a quarter of the total trauma patients in this group. Table 1; Fig. 1 reflect the 11-year geriatric trauma trends: the total injury rate for the older group (> 65 years) was 24 per 10,000 inhabitants compared to 10 per 10,000 in the younger group (55–64 years). 35% of geriatric injuries occurred at home, and 46% happened on the street. The injury rate showed a decreasing trend over the years in both age groups (Table 1). The most common MOI was falls (46%), followed by road traffic injuries [RTIs] (41%), like occupants in MVC and pedestrian crashes (Table 2). The most injured regions were the chest (45%), lower limbs (31%), head (28%), and abdomen (11%), in contrast to fewer upper extremity injuries. The median length of hospital stay was six days (range 1-182). The mean ISS was 13 ± 9. The overall in-hospital mortality was 8%.

**Table 1:** Number and rate of hospitalized older trauma patients across the years 2010–2021 in Qatar

Year	> 65 Years	55–64 Years	Estimated population ages 65 and above in Qatar	Estimated population between ages 55–64 in Qatar	Rate of injury per 10,000	
					≥65 years	55–64 years
2010-11*	57	78	13,265	-	42.9	-
2011-12	55	84	15,229	59,782	36.1	14.05
2012-13	49	74	17,548	65,467	27.9	11.3
2013-14	40	72	21,120	72,158	18.9	10.0
2014-15	46	80	22,820	82,487	20.1	9.7
2015-16	54	100	25,619	93,036	21.07	10.7
2016-17	56	93	29,433	103,288	19.02	9.0
2017-18	69	77	33,572	101,167	20.1	7.6
2018-19	81	90	38,111	102,125	21.2	8.8
2019-20	89	93	43,137	104,613	20.6	8.9
2020-21**	95	94	48,665	107,815	19.5	8.7
Total/Average	**691**	**935**	**308,519**	**891,938**	**24.3**	**9.9**

* Duration is 20 months (May 2010 to December 2011), ** Duration is 20 months (January 2020 to April 2021)

**Table 2:** Demographics, clinical characteristics of the study population

Variables	Values (*N*; %)	Variables (*N*; %)	Values (*N*; %)
**Age**, **Years; mean ± SD**	65.5 ± 9.5	**Comorbidities**	
**Gender**		Diabetes	677 (41.6%)
Male	1341 (82.5%)	Hypertension	759 (46.7%)
Female	285 (17.5%)	Heart disease	296 (18.2%)
Type of Protective Devices* for passengers		Cancer	22 (1.4%)
Seat Belt	150 (9.2%)	CVA	68 (4.2%)
Airbag	14 (0.9%)	Hypothyroidism	12 (0.7%)
Seat belt + Airbag	50 (3.1%)	Kidney Disease	48 (3%)
Helmet	17 (1.0%)	Dementia/Alzheimer/depression	26 (1.6%)
**Location of Injury**		Obesity	10 (0.6%)
Home	560 (35.2%)	COPD/asthma	49 (3.0%)
Street	741 (46.5%)	**Associated Injuries**	
Farm	6 (0.4%)	Head Injury	448 (27.6%)
Recreational	27 (1.7%)	Chest Injury	735 (45.2%)
Work-related	197 (12.4%)	Abdominal Injury	193 (11.9%)
Public place	32 (2.0%)	Leg Injury	505 (31.1%)
Others	29 (1.8%)	Arm Injury	2 (0.1%)
Unknown	34 (2.1%)	**Complications**	
**Mechanism of Injury**		ARDS	13 (0.8%)
Fall	754 (46.4%)	Acute renal impairment	33 (2%)
MVC	429 (26.4%)	Pneumonia	76 (4.7%)
Pedestrian	245 (15.1%)	Sepsis	50 (3.1%)
Fall of heavy object	54 (3.3%)	Thromboembolic events	12 (1.2%)
Assault	24 (1.5%)	CVA	6 (0.6%)
MCC	39 (2.4%)	Wound Infections	36 (2.2%)
ATV	18 (1.1%)	Urinary tract infection	52 (3.2%)
Machinerelated injury	15 (0.9%)	Pressure Ulcer	18 (1.1%)
Burn	6 (0.4%)		
Self-Inflicted	5 (0.3%)		
Others	37 (2.8%)		

Abbreviations: MVC: Motor vehicle crash; ATV: All-terrain vehicle; MCC: Motorcycle crash; CVA: Cerebrovascular accident, COPD: Chronic obstructive pulmonary disease; ARDS: Acute respiratory distress syndrome

Figure 1 shows the trends of trauma hospitalization by year and gender (the 2010–2011 period included only 20 months, and the 2020–2021 period included 16 months). Injured females represented a minority of this population during the study period. The most common mechanisms of injury were Falls (46%), MVCs (26%), and pedestrian crashes (15%). For the MVC group, the reported use of protective devices was seat belt in 150 (9.2%) patients, airbag 14 (0.9%), seat belt + airbag 50 (3.1%), and helmet 17 (1.0%) [Table 2].

**Fig. 2:**
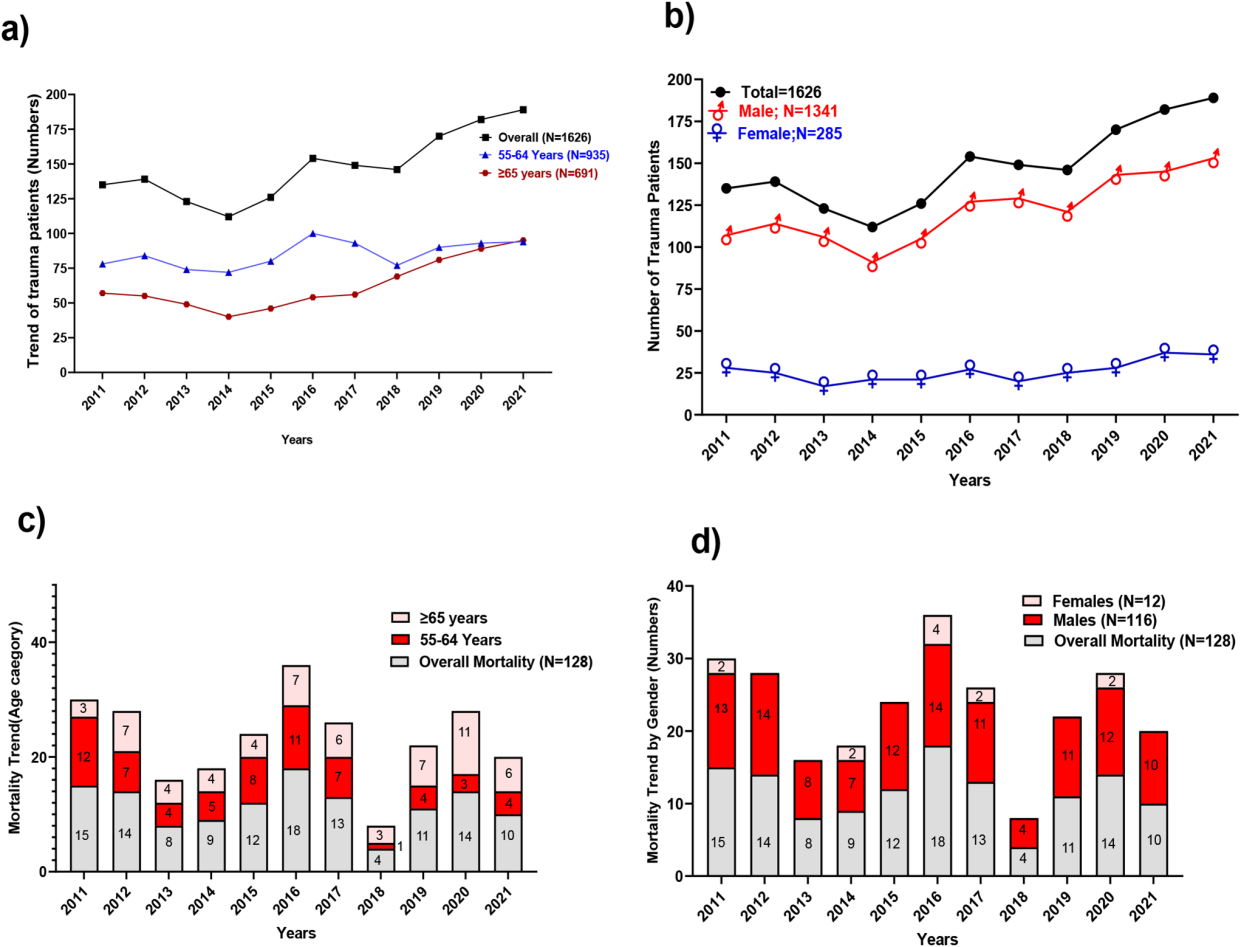
(**a**) The trend of trauma patients aged 55–64 and ≥ 65 years using trauma registry data over the study period (2010–2021). (**b**) The trend of trauma patients stratified by Gender. (**c**) The mortality trend of the studied population stratified by age category. (**d**) The mortality trend of the studied population stratified by Gender

Males had more head, chest, and abdominal injuries and higher mortality than females (9% vs. 4%).

The most common interventions were intubation (17%), fracture fixation (14%), and chest tube insertions (11%), while there were few cranial surgeries (3%), laparotomies (3%), and thoracotomies. Blood transfusion was needed in 20%, while massive transfusion activation protocol (MTP) (transfusion of > 10 units over 24 h) was necessary in 2%. Thirty percent of the cohort required ICU admission. The median overall length of stay was six days, and the median ICU LOS (length of stay) was four days. In-hospital complications included pneumonia (4.7%), urinary tract infection (UTI) (3.2%), and sepsis (3.1%). At the same time, rehabilitation and discharge to long-term care facilities happened in 13%.

A comparison between age groups (55–64 vs. ≥ 65 years) is shown in Table 3. The older patient group had more comorbidities (diabetes mellitus, hypertension, coronary artery disease, stroke, dementia, and cancer), a higher percentage of females (27% vs. 10.5%), a higher rate of frailty (73% vs. 50%) and more hospital events in terms of complications (renal failure, sepsis, and UTI) (Table 3). These findings correspond to an increase in the need for renal replacement therapy, and the overall length of stay. On the other hand, the younger group had more severe injuries in terms of higher AIS as well as ISS and more head injuries and chest and abdominal injuries than the older group. Also, they had more road traffic injuries (RTIs), higher SI, and a need for resuscitation (fluids, blood, and blood products, including MTP activations). Nevertheless, no significant differences in ISS, blood transfusion, ICU LOS, ventilator days, and mortality were noted between these two groups. Figure 1B shows that Falls were the leading MOI in the older age group, while RTIs were the leading MOI in the younger group.

**Table 3:** Characteristics and outcomes of older adults (aged ≥ 65 and 55–64 years.) Treated at the Trauma Center from 2010–2021, stratified by Age Category

Variables	55–64 years. (*n* = 935)	≥ 65 years. (*n* = 691)	*P* value	Variables	55–64 years. (*n* = 935)	≥ 65 years. (*n* = 691)	*P* value
**Age**; mean and SD Median and range	58.84 ± 2.83 58(55–64)	74.40 ± 7.96 71(65–96)	0.001	**Interventions**			
**Gender**				Chest Tube	109 (11.7%)	65(9.4%)	0.168
Male	837 (89.5%)	504 (72.9%)	0.001	Intubation	169 (18.1%)	111(16.1%)	0.319
Female	98 (10.5%)	187 (27.1%)		Exploratory laparotomy	39 (4.2%)	8 (1.2%)	0.001
Documented use Protective Devices (214/429)**	144 (51.8%)	70 (46.4%)	0.28	Tracheostomy	35 (3.7%)	35(5.1%)	0.120
**Comorbidities**				Orthopedic surgery	137 (14.7%)	86 (12.4%)	0.215
Diabetes Mellitus	326 (34.9%)	351 (50.8%)	0.001	Dialysis	12 (1.3%)	26 (%)	0.001
Hypertension	328 (35.2%)	430 (62.2%)	0.001	Crystalloids	692 (74%)	402 (58.2%)	0.001
Heart Disease	110 (11.8%)	186 (26.9%)	0.001	**Blood transfused**	170 (18.2%)	151 (21.9%)	0.068
Malignancy	6 (0.6%)	16 (2.3%)	0.004	**FFP**	63 (6.7%)	49 (7.09%)	0.843
CVA	19 (2.0%)	65 (9.4%)	0.001	**Platelets Transfusion**	52 (5.6%)	52 (7.5%)	0.124
**Associated Injuries**				**MTP activation**	28 (33.4%)	9 (1.3%)	0.028
Head Injury	237 (74.7%)	211 (69.5%)	0.012	**ISS**	13.04 ± 9.44	12.19 ± 7.86	0.058
Chest Injury	453 (48.6%)	281(40.7%)	0.001	**RTS**	7.54 ± 0.98	7.65 ± 0.73	0.016
Abdominal Injury	140 (15.0%)	53 (7.7%)	0.001	**Shock Index**	0.65 ± 0.23	0.61 ± 0.34	0.001
Arm Injury	1 (0.1%)	1 (0.1%)	0.669	**Outcomes**			
Leg Injury	276 (29.5%)	229 (33.1%)	0.066	Ventilatory Days (*n* = 256)	4 (1–97) *n* = 153	7 (1-144) *n* = 103	0.092
**Hospital Complications**				Total HLOS	13 (1-142)	15.5 (1-182)	0.001
ARDS	7 (0.7%)	6 (0.9%)	0.500	ICU LOS	4 (1-142)	5 (1-144)	0.134
Acute kidney injury	12 (1.3%)	21 (3.0%)	0.011	Mortality	66 (7.1%)	62 (9.0%)	0.163
Pneumonia	43 (4.6%)	33 (4.8%)	0.479				
Sepsis	21 (2.2%)	29 (4.2%)	0.029				
VTE	6 (0.6%)	6 (0.8%)	0.578				
Urinary tract infection	20 (2.1%)	32 (4.6%)	0.006				
Wound Infection	21(2.2%)	15 (2.2%)	0.919				

Abbreviations: CVA: cerebral vascular accident; ARDS: Acute respiratory distress syndrome; VTE: Venous thromboembolism; FFP: Fresh frozen plasma; MTP: Massive Transfusion Protocol; ISS: Injury severity Score; RTS: Revised trauma score; HLOS: Hospital length of stay; ICU LOS: Intensive care unit length of stay

**Fig. 3:**
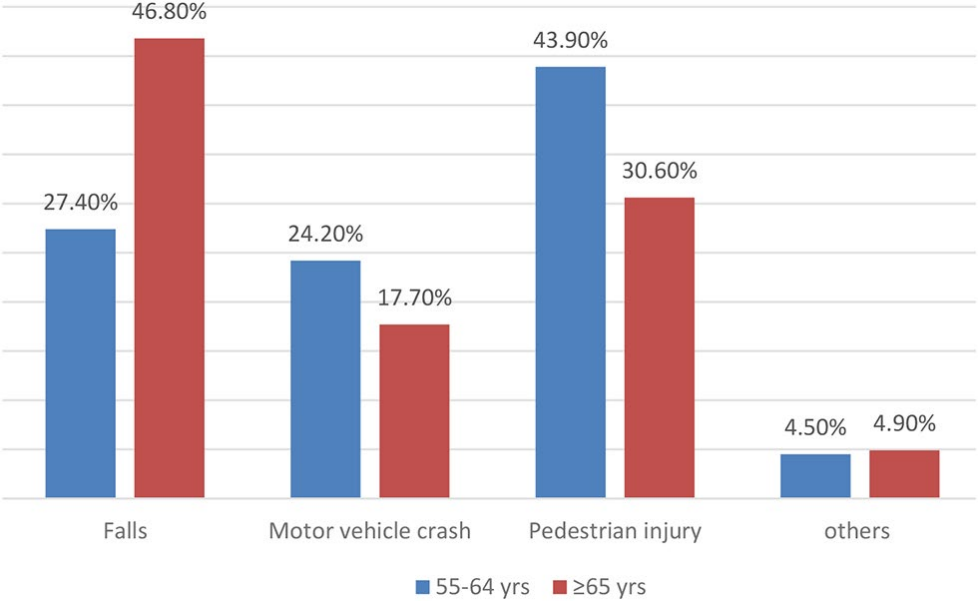
The proportion of mortality in each mechanism of injury in the two study groups (*p* = 0.001)

Age ≥ 65 years: A sub-analysis of patients aged 65 years and older revealed age-specific trends in patient numbers, hospital length of stay (HLOS), and mortality rates. Patients were grouped by decades: The median HLOS was 6 days (range: 1–166) for those aged 65–74 years (*n* = 387), 8 days (range: 1–182) for 75–84 years (*n* = 234), 8 days (range: 1–144) for 85–94 (*n* = 61) years, and 1 day (range: 1–147) for patients over 95 years (*n* = 9). The highest mortality was observed in patients aged > 95 years (44.4%) who also had the shortest median HLOS. Figure 3 shows the mortality in each age group according to the MOI. Falls represented the higher proportion of mortality among the elder group 46.8% (29/62) vs. 27.4% (15/66) whereas pedestrian injury and MVC were more evident causes of death among the younger group.

Table 4 compares demographics and clinical variables by gender across the entire sample. On average, female patients constituted a larger proportion of the older age group, were more frequently injured due to falls, and exhibited higher frailty (72% vs. 60%). Though, in absolute numbers, even among locals, males dominated. More leg injuries, moderate ISS, and more had home disposition and lower mortality than males despite being frailer, which may reflect the higher severity of trauma observed in males, nullifying the frailty effect. In contrast, males had more head injuries and chest and abdominal injuries. More numbers are in the three other ISS categories, i.e., severe, profound, and mild ISS.

**Table 4:** Mechanism of injury, injury severities, complications, and outcome based on gender

Variables (*N*; %)	Total (*N* = 1626)	Male (*N* = 1341)	Female (*N* = 285)	*P* value
**Age; mean ± SD**	65.5 ± 9.5	64.4 ± 8.9	70.3 ± 10.5	0.001
**Mechanism of Injury; N (%)**				0.001
Fall	754 (46.4%)	569 (42.4%)	185 (64.9%)	
Traffic-Related injuries	468 (28.8%)	406 (30.3%)	62 (21.8%)	
Pedestrian	245(15.1%)	225(16.8%)	20 (7.0%)	
Fall of heavy objects	54 (3.3%)	50(3.7%)	4(1.4%)	
Others	105 (6.5%)	91 (6.8%)	15 (4.9%)	
**5-Factor Modified Frailty Index**				
0	654(40.2%)	575 (42.9%)	79 (27.7%)	0.001
1	460 (28.3)	375 (28.0%)	85 (29.8%)	
2	326(20.0%)	242 (18.0%)	84 (29.3%)	
3–5	186(11.4%)	149 (11.1%)	37(13.0%)	
**Associated Injuries; N (%)**				
Head Injury	448 (27.6%)	391 (29.2%)	57 (20.0%)	0.002
Chest Injury	735 (45.2%)	626 (46.6%)	109 (38.2%)	0.011
Abdominal Injury	193 (11.9%)	182 (13.5%)	11 (3.9%)	0.001
Leg Injury	505 (31.1%)	397 (29. %)	108 (37.9%)	0.007
**Injury severity score (ISS); N (%)**				
1–8 (Mild)	388 (24.3%)	313 (23.8%)	752 (6.8%)	0.001
9–15 (Moderate)	784 (49.1%)	622 (47.2%)	162 (57.9%)	
16–24 (Severe)	259 (16.2%)	231(17.5%)	28 (10.0%)	
> 24 (Profound)	166 (10.4%)	151 (11.5%)	15 (5.4%)	
**Revised Trauma Score (RTS); N (%)**				0.001
1-4.9	61(4.1%)	59 (4.8%)	2 (0.8%)	
≥ 5	1422 (95.9%)	1160 (95.2%)	262 (99.2%)	
**Discharge Disposition; N (%)**				
Home	1319 (81.5%)	1070 (80.1%)	249 (87.7%)	0.01
Rehabilitation	79 (4.9%)	73 (5.5%)	6 (2.1%)	
Long Term	18 (1.1%)	14 (1.0%)	4 (1.4%)	
Transfer	74 (4.6%)	61 (4.6%)	13 (4.6%)	
**Outcome; N (%)**				0.01
Alive	1498 (92.1%)	1225 (91.3%)	273 (95.8%)	
Dead	128 (7.9%)	116 (8.7%)	12 (4.2%)	

Table 5 compares survivors with non-survivors. Non-survivors were older and had significantly higher rates of cardiac and renal comorbidities. They also had more associated head and abdominal injuries, which contributed to the higher mortality observed both in younger non-frail patients and those who were severely frail. Also, a higher percentage of patients had a lower GCS, higher ISS, shock index, and a lower revised trauma score: more required blood transfusion, FFP, platelets, and MTP activation. Fatalities also had more hospital complications, including pneumonia, sepsis, acute respiratory distress syndrome, pressure ulcers, and significantly more ventilator days and longer ICU and total LOS. The deceased had an average age of 62 years compared to an average age of 59 years for the survivors, had more head injuries, and received more massive transfusions (MTP). Figure 2 depicts the mortality stratified by age and gender, with male predominance. Figure 1 displays the age trends over the years and the mechanism of injuries across the two groups of the elderly.

**Table 5:** Characteristics of patients based on outcome (mortality)

Variables	Survivors (*n* = 1498)	Non-Survivors (*n* = 128)	*P*- value
**Age; (Mean**, **SD)**	59.3 ± 2.08	62.0 ± 9.9	0.018
**Comorbidities**			
Hypertension N, (%)	709 (47.3%)	50 (39.06%)	0.079
Diabetes Mellitus N, (%)	630 (42.1%)	47 (36.7%)	0.263
Heart Disease N, (%)	264 (17.6%)	32 (25.0%)	0.043
CVA; N, (%)	61 (4.1%)	7 (5.5%)	0.486
Kidney Disease; N, (%)	39 (2.6%)	9 (7.03%)	0.01
CA N, (%)	18 (1.2%)	4 (3.1%)	0.088
Asthma; N, (%)	39 (2.6%)	2 (1.6%)	0.767
COPD; N, (%)	7 (0.5%)	1 (0.8%)	0.482
**5-Factor Modified Frailty Index**			
0	586(39.1%)	68(53.1%)	0.001 for all
1	441(29.4%)	19(14.8%)	
2	308 (20.6%)	18(14.1%)	
3–5	163(10.9%)	23(18.0%)	
**Associated Injuries**			
Head Injury; (N, (%)	364 (24.3%)	84 (65.6%)	0.001
Chest Injury N, (%)	673 (44.9%)	62 (48.4%)	0.444
Abdominal Injury; N, (%)	168 (11.2%)	25 (19.5%)	0.005
Leg Injury; N, (%)	438 (29.2%)	37 (28.9%)	0.584
Arm Injury; N, (%)	1 (0.06%)	1 (0.8%)	0.151
**Associated Complications**			
Acute Kidney Injury	9 (0.60%)	24 (18.8%)	0.001
Pneumonia	57 (3.8%)	19 (14.8%)	0.001
Sepsis	37 (2.5%)	13 (10.1%)	0.001
Urinary Tract Infections	42 (2.8%)	10 (7.8%)	0.006
Acute Respiratory Distress Syndrome	7 (0.5%)	6 (4.7%)	0.001
Pressure Ulcer	13 (0.9%)	5 (3.9%)	0.001
Wound Infection	33 (2.2%)	3 (2.3%)	0.758
Pulmonary Embolism	7 (0.5%)	2 (1.6%)	0.154
**Blood Product Administration**			
Blood transfusion	258 (17.2%)	63 (49.2%)	0.001
Fresh Frozen Plasma	74 (4.9%)	38 (29.7%)	0.001
Platelets	65 (4.3%)	39 (30.5%)	0.001
**Massive Transfusion Protocol activation**	18 (1.2%)	19 (14.8%)	0.001
**Glasgow Coma Score at Scene; mean ± SD**	8.3 ± 5.5	5.0 ± 1.4	0.001
**Injury Severity Score; mean ± SD**	34.3 ± 10.9	35 ± 11.3	0.001
**Revised Trauma Score; mean ± SD**	5.7 ± 1.5	4.7 ± 0.37	0.001
**Shock Index; mean ± SD**	0.63 ± 0.27	0.83 ± 0.42	0.001
**Intensive Care Unit LOS; median (range)**	4 (1-150)	6 (1-144)	0.010
**Total Length of Stay; median (range)**	6 (1-182)	5 (1-144)	0.001
**Ventilatory Days; median (range)**	6 (1-100)	4 (1-144)	0.050

**Fig. 4:**
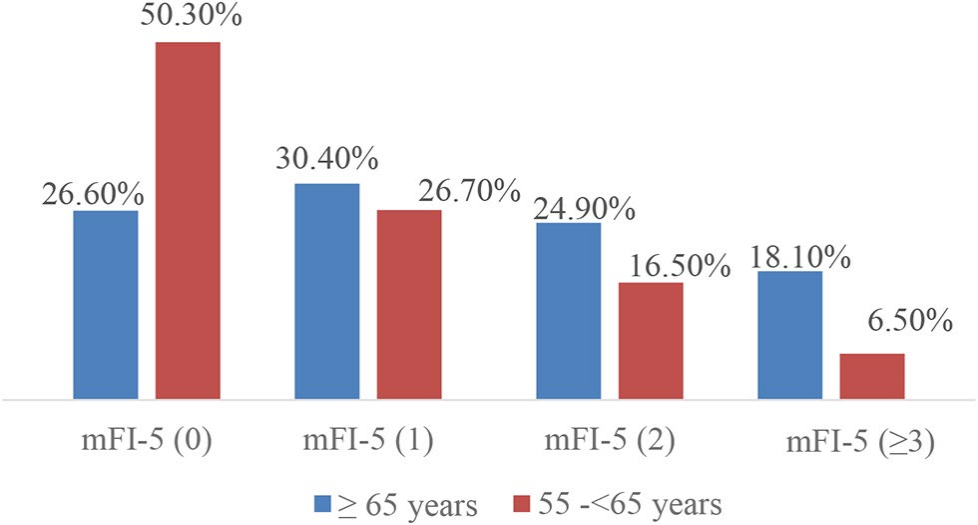
The 5-factor modified frailty index (mFI-5) based on the age group

Table 6 shows that moderate and severe frailty were more prevalent among females, although males dominated across all groups due to the demographic characteristics of the country. Higher frailty grades were associated with home-related falls and head injuries. In contrast, patients with no or mild frailty, who were more mobile, were more frequently injured on the street, resulting in more leg injuries. However, the difference was not statistically significant. Notably, severely frail patients had a higher usage of anticoagulation, a potential confounding factor. Moreover, patients aged 65 years or older are more likely to have higher frailty scores, as indicated by higher percentages in the mFI-5 (1), (2), and (≥ 3) categories (Fig. 4).

**Table 6:** Patient’s demographic and clinical characteristics stratified by 5-Factor modified Frailty Index

Variables	0 (No Frailty) (*N* = 654)	1(Mild Frailty) (*N* = 460)	2(Moderate Frailty) (*N* = 326)	3+ (Severe frailty) (*N* = 186)	*p*-value
Age	63.1 ± 9.4	65.9 ± 9.4	66.9 ± 8.5	70.2 ± 9.5	0.001
**Gender**					
Male	87.9%	81.5%	74.2%	80.1%	0.001
Female	12.1%	18.5%	25.8%	19.9%	
**Location of injury**					
Home	156(24.6%)	172(37.7%)	144(45.1%)	88(48.1%)	0.001 for all
Street	315(49.7%)	204(44.7%)	143(44.8%)	79(43.2%)	
Farm	4(0.6%)	1(0.2%)	1(0.3%)	0(0.0%)	
Recreational	17(2.7%)	6(1.3%)	3(0.9%)	1(0.5%)	
Workplace	122(19.2%)	55(12.1%)	15(4.7%)	5(2.7%)	
Public Place	5(0.8%)	9(2.0%)	10(3.1%)	8(4.4%)	
Others	15(2.4%)	9(2.0%)	3(0.9%)	2(1.1%)	
**Mechanism of injury**, **n (%)**					
Fall	262(40.1%)	226(49.1%)	167(51.2%)	99(53.2%)	0.001 for all
Traffic related injuries	175(26.8%)	132(28.7%)	104(31.9%)	57(30.6%)	
Pedestrian	129(19.7%)	60(13.0%)	38(11.7%)	18(9.7%)	
Fall of heavy object	33(5.0%)	14(3.0%)	3(0.9%)	4(2.2%)	
Others	55(8.4%)	28(6.1%)	14(4.3%)	8(4.3%)	
**Physiological parameters**					
Pulse rate	84.5 ± 17.7	85.2 ± 17.6	87.3 ± 17.7	84.5 ± 18.1	0.135
Respiratory Rate	19.4 ± 3.4	19.8 ± 4.3	20.0 ± 6.7	20.1 ± 5.7	0.244
SBP	136.8 ± 25.7	140.8 ± 27.4	146.2 ± 27.9	140.2 ± 32.1	0.001
DBP	80.3 ± 14.7	80.5 ± 16.1	81.0 ± 16.8	75.5 ± 18.2	0.001
Oxygen Saturation	98.1 ± 4.1	97.9 ± 2.5	97.6 ± 5.7	97.4 ± 3.5	0.104
Scene GCS	13.5 ± 3.8	14.1 ± 2.8	14.1 ± 2.7	14.1 ± 2.7	0.001
ISS	12.8 ± 9.7	12.0 ± 7.6	12.1 ± 7.7	14.8 ± 9.9	0.002
RTS	7.5 ± 1.1	7.6 ± 0.8	7.7 ± 0.7	7.6 ± 0.8	0.146
Anticoagulants	0(0.0%)	13(2.8%)	2 (0.6%)	11(5.9%)	0.001
**Injury Types**					
Head Injury	172(26.3%)	107 (23.3%)	89(27.3%)	80(43.0%)	0.001
Chest injury	299(45.7%)	203 (44.1%)	154(47.2%)	79(42.5%)	0.708
Abdominal Injury	92(14.1%)	52 (11.3%)	30 (9.2%)	19(10.2%)	0.118
Arm Injury	1(0.2%)	0(0.0%)	1 (0.3%)	0(0%)	0.628
Leg Injury	210(32.1%)	153 (33.3%)	94 (28.8%)	48(25.8%)	0.210

DBP: Systolic Blood Pressure; GCS: Glasgow Comma Scale; ISS: Injury Severity Score; ISS: Injury severity score; RTS: Revised trauma score

Table 7 indicates that 73% of the study cohort were frail. Among the older group, 30% were mildly frail, 25% moderately frail, 18% severely frail, and the remaining were not frail. In the younger group, 50% were frail (27% mildly frail, 16.5% moderately frail, and 6.5% severely frail), while the other half were not frail according to the mFI-5 tool. Higher frailty scores correlated with increased complications, particularly acute kidney injury (AKI), pneumonia, and urinary tract infections. This also led to longer hospital and ICU stays, a greater need for ventilator support, and longer ventilator days.

**Table 7:** Patient outcomes stratified frailty [5-Factor modified Frailty Index]

Variables	0 (No Frailty) (*N* = 654)	1(Mild Frailty) (*N* = 460)	2 (Moderate Frailty) (*N* = 326)	3+ (Severe frailty) (*N* = 186)	*p*-value
**Complications**, ***n*** (**%**)					
ARDS	4(0.6%)	4(0.9%)	2(0.6%)	3(1.6%)	0.570
Acute renal impairment	9(1.4%)	7(1.5%)	4 (1.2%)	13(7.0%)	0.001
Pneumonia	20(3.1%)	19(4.1%)	23(7.1%)	14(7.5%)	0.009
Sepsis	12(1.8%)	15(3.3%)	14(4.3%)	9(4.8%)	0.072
Deep vein thrombosis	2(0.3%)	0(0.0%)	0 (0.0%)	1(0.5%)	0.357
Pulmonary Embolism	3(0.5%)	1(0.2%)	4(1.2%)	1(0.5%)	0.291
Wound infection	9(1.4%)	12(2.6%)	8 (2.5%)	7(3.8%)	0.203
Pressure ulcer	4(0.6%)	2(0.4%)	8(2.5%)	4(2.2%)	0.014
Urinary tract infection	9(1.4%)	16(3.5%)	21(6.4%)	6(3.2%)	0.001
**Interventions**, **n (%)**					
FAST	573(87.6%)	420(91.3%)	292 (89.6%)	162(87.1%)	0.206
Intubation	112(17.1%)	68(14.8%)	58(17.8%)	42(22.6%)	0.125
Exploratory laparotomy	20(3.1%)	13(2.8%)	10 (3.1%)	4(2.2%)	0.925
Tracheostomy	16(2.4%)	17(3.7%)	22(6.7%)	15(8.1%)	0.001
Dialysis	6(0.9%)	8(1.7%)	11 (3.4%)	13(7.0%)	0.001
Crystalloid	447(68.3%)	312(67.8%)	213 (65.3%)	122 (65.6%)	0.753
Blood transfusions	111(17.0%)	84(18.3%)	70(21.5%)	56 (30.1%)	0.001
Fresh frozen plasma	32(4.9%)	34(7.4%)	19 (5.8%)	27 (14.5%)	0.001
Platelets	31(4.7%)	26(5.7%)	22 (6.7%)	25 (13.4%)	0.001
MTP Activation	19(2.9%)	15(2.6%)	5 (1.5%)	1(0.5%)	0.193
Home discharge	521(89.7%)	387(88.2%)	274(89.3%)	137(84.0%)	0.229
Rehabilitation	24(4.1%)	24(5.8%)	18(6.2%)	13(8.7%)	
HLOS, median (Range)	5(1-125)	6(1-175)	8(1-144)	10(1-182)	0.001
ICU LOS, median (Range)	3 (1–48)	4(1-123)	5(1-144)	5(1-150)	0.001`
Ventilator, (yes) n (%)	94 (14.4%)	65(14.1%)	56(17.2%)	41(22.0%)	0.048
Ventilator days, median (Range)	3(1–28)	5(1-104)	8(1-144)	6(1-100)	0.007
**Mortality**, **n (%)**	68(10.6%)	19(4.1%)	18(5.5%)	23(12.4%)	0.001

ARDS: Acute respiratory distress syndrome; CVA: Cerebrovascular Accident; FAST: Focused Assessment with Sonography in Trauma; HLOS: Hospital length of stay; ICU: Intensive Care Unit

Additionally, frail patients required more blood transfusions, though massive transfusion protocol (MTP) activation was not significantly different between frail and non-frail patients. Mortality was higher among severely frail and non-frail patients. Furthermore, severely frail patients had a greater need for tracheostomy, reflecting longer ventilation requirements, as well as dialysis due to a higher incidence of AKIs. Non-home discharge rates were significantly higher in the severe frailty group compared to the moderate and non-frailty groups.

In multivariable regression analysis, severe frailty was a significant predictor of longer hospitalization (adjusted OR, 1.83; 95% CI 1.183–2.833; *P* = 0.007) (Table 8).

**Table 8:** Multivariable regression analysis for predictors of hospital length of stay more than a week

Variable	*P* value	Odds ratio	95% confidence interval	
Age	0.462	1.006	0.991	1.020
Gender (female)	0.001	1.791	1.285	2.497
Injury severity score	0.001	1.049	1.029	1.069
Acute renal impairment	0.158	0.464	0.160	1.346
GCS at the ED	0.394	1.020	0.975	1.066
Frailty Score Index (FSI)	0.004			
FSI (1)	0.040	1.377	1.015	1.867
FSI (2)	0.002	1.731	1.227	2.443
FSI (≥ 3)	0.007	1.830	1.183	2.833
Shock index	0.384	0.795	0.475	1.332
Blood transfusion	0.001	6.001	4.113	8.756
Pneumonia	0.001	12.427	3.692	41.826

## Discussion

We have analyzed an eleven-year geriatric (defined by age ≥ 55 years) trauma population treated at the national trauma center of Qatar, a Middle Eastern state with nearly 3 million people, with the majority living in urban areas (93–96%). These patients comprise one out of eleven trauma admissions (9.2%), with a male predominance (80%) and a mean ISS of 13. Falls are the leading mechanism of geriatric trauma in Qatar (46%), with one-third happening at home (35.2%), but the road was the leading location of injury (46.5%), and RTIs came in as the second leading MOI (45%). The chest was the most injured region, and one in twelve (8%) of them succumbed to their injuries.

There is no consensus on the age that defines a geriatric population, although 65 years has been the traditional cutoff point [9–12]. However, many recent reports note significantly higher mortality for trauma patients, starting at ages as low as 45–55 years [4, 9, 11, 24]. “Rather than having a derogatory connotation, the term geriatric represents the statistically significant inflection point in patients’ morbidity and mortality for a given injury compared with a younger patient” [25]. This other point is the rationale for collecting our data a decade earlier than the traditional, most-referred-to cutoff age of 65.

The mortality rate in our study analysis (8%) was lower than that of reports from many Western countries [9, 12, 15, 26, 27]. Mangram et al. reported that a one-year study with small a sample size of 393 patients after the institution of a dedicated geriatric unit (DGU) with a multidisciplinary team led by trauma surgeons compared to an earlier control sample of 280 patients. The majority were blunt injuries related to falls. There was a lower mortality (3.8% vs. 5.7%, p = NS) in those who were treated in the DGU [28]. An earlier National trauma databank-based study over ten years reported that the elderly (> 60 years) have a 5-fold increase in mortality with minor ISS (0–9), whereas they have a 4-fold increase in mortality with major ISS (10–15) [29].

However, closer attention must be paid to the ‘expected’ findings when comparing survivors with non-survivors [Table 5]. Non-survivors are statistically sicker or more severely injured than the survivors. However, mortality, ICU LOS, and ventilator days were comparable between both age groups. Apparently, the significantly higher prevalence of co-morbidities in the older age group is offset by the higher percentage of younger patients with head, chest, and abdominal injuries. Traditional predictors of trauma mortality, like age, co-morbidities, chest, abdominal, and head injuries, and even ISS, should be viewed with a new lens due to the complex interaction of both age and mechanism-dependent factors. Older patients are ‘expected’ to have more co-morbidities and suffer from more complications [29]. Patients from the younger age group, however, are more likely to be victims of the transfer of higher energy from RTIs, ergo incurring more injuries to the head, chest, and abdomen and requiring more intervention. This evidence supports the ‘new’ definition of geriatric trauma because while their paths may differ, all of these patients have similar outcomes. Therefore, all of these trauma patients, 55 years and older, should be selectively cared for by multi-disciplinary teams who will implement clinical trauma protocols that are best suited for this unique trauma population; furthermore, we might consider age as a criterion for level of trauma activation as reported before that activation level impacts outcomes [28, 29] and age of 55 and above is an independent predictor of multiorgan failure [30].

When applying the 5-Factor Modified Frailty Index, clearer patterns of injury emerge. The severely frail (score ≥ 3) are more likely to suffer from low-energy falls from standing at home, while the least frail (score = 0) are victims of high-energy transfer RTIs while on the street. Despite statistically significant differences between physiological parameters, head injuries, complications, and interventions, the mortality rates for the non-frail and the most severely frail were not significantly different [*p* = 0.49] in Table 7.

In an earlier U.S. report (2008), Geriatric trauma patients, those 65 years and older, comprised 14% of trauma-related ED visits, with an increasing trend [25, 27, 31, 32]; this is projected to reach 20% in 2050 [33]. Florio et al. reported a higher percentage (up to 20%) in Europe [28]. Both are higher than our cohort-reported proportion of 9.2%. Notice that we applied a lower cutoff of 55, which reflects a much lower incidence in our population compared to the rest of the published world data. This low figure may represent an underestimation simply because our data represent only those who needed hospital admission and because the ratio of Qatar’s population is much lower than that of the U.S. or Europe.

Frailty, or the reduced physiological reserve commonly associated with aging, is often linked to a range of comorbid conditions. This relationship is captured by tools such as the modified Frailty Index-5 (mFI-5) used in this study [18]. The modified mFI-5 index used in this study is effective across all trauma patients, regardless of age, though its prevalence and severity increase with older age groups. This corresponds to observed adverse outcomes, including higher rates of mortality, morbidity, longer hospital stays, unplanned events, and non-home discharges, as also reported in other surgical populations like the general surgery studies [31, 32, 34–36]; arthroplasty [37], multiple orthopedic trauma [38]; and more recently in the trauma population [19, 39]. Our cohort reflects a common phenomenon, with severely frail patients comprising approximately 25% of the total group.

Qatar’s population is aging like the rest of the world, with [24–26, 33, 40–42], reflecting better health care and healthier, active lifestyles. In this cohort, we documented a recent increase in both selected age groups, those ≥ 65 and those 55–64 years old. The incidence of injuries per 10,000 has increased among the elderly compared to the younger age group (55–64), making them a particularly high-risk group, conforming to published evidence. The global estimate is that people > 65 will represent a fifth of the world population, especially in developed countries with better life expectancy and care [26, 33, 41, 42].

Interestingly, our data showed a male predominance across the entire age spectrum. This male dominance is well known for younger trauma patients but not for geriatric trauma. On the other hand, the percentage of females in the classic geriatric age group (≥ 65y) is at a near-normal sex distribution. Higher levels of physical activity and a predominance of male drivers could explain the persistent male predominance in the geriatric trauma population, while home falls are more in elderly females [42]. Nevertheless, another possible explanation is that patients with only ‘hip’ fractures, a classic female majority population, are not included in this analysis because they are not admitted to the trauma service or captured in the trauma registry. Nevertheless, previously published local data on hip fractures in Qatar do not support this, as male predominance is also observed in the hip fracture population [43].

The study revealed a near-exclusive blunt mechanism of injury (penetrating was < 0.5%), with most falling from a standing position at ground level. The literature reports the similar pattern. Though with higher percentages [1, 2, 5, 15, 16, 26, 42, 44, 45]. The underlying physiological changes, comorbidities, and treatments may all affect gait stability and balance, putting the elderly at a higher risk for this mechanism even at the ground level and in homes [27, 44–46]. Assaults, self-inflicted injuries, and elder abuse were very few, in contrast to a relatively higher percentage in Western data [46, 47].

The home was the most reported location for the injuries, reflecting that most are in post-retirement and partly explaining the absence of the weekend and off-hours effects in this cohort. A similar home location for elder falls dominates reports from the rest of the world [5, 48–51]. Furthermore, the Al-Ain Hospital in the UAE showed that falls represented 55% of the observed mode of injury and had more falls in females than males [52]. The falling percentage was 47% overall; our lower age limit might explain the lower rate in males and the higher number of females involved in this mechanism (64% compared to 42% males), a finding that compares to the rest of the world. A prior study from the USA showed that the risk of fall increased with age, with an OR 1.52 for age 70–79 and an OR 3.40 for ≥ 80, whereas females fell 1.2 times more [53]. At the same time, the second mechanism was the MVC in a quarter, which is like other international reports [42, 51].

The chest was the most injured region in this cohort; Ferrah et al. reported a similar predominance in chest injuries among elderly trauma patients, who attributed that to the liberal use of computed tomography in recent data compared to historical data [54].

Geriatric injured patients represent 20% of TICU admissions in Spain [53], less than we observed in this cohort (30%). The higher percentage of critical care admissions may be due to our cohort’s higher incidence of TBI and medical comorbidities; it may also reflect more generous admission criteria, leaning toward safety and the fact that the ICU is under the trauma service management and leadership. There was no significant difference between the younger (55 - <65) and ≥ 65y in mortality and other clinical outcomes except for the total hospital LOS, which supports our newly designated cutoff age of 55years going with previously mentioned resources suggesting this new definition for geriatrics [9, 11, 12].

The most common discharge destination was home (81.5%). Around a fifth were discharged to a long-term rehabilitation facility, reflecting that some geriatric trauma patients cannot immediately regain their pre-injury functional status [53]. The relatively low percentage of rehab disposition compared to other higher rates can be partially explained by the lower capture point of 55 and may reflect different social support systems.

This report indirectly reflects the higher cost of caring for this population, considering the higher length of hospital and ICU stay with a frequent need for operative orthopedic fixation in the elderly, the associated comorbidities and their treatments, and the need for rehabilitation and long-term services [11, 51].

Although the retrospective registry data collection was well maintained, this study is not without limitations. The retrospective design, the documentation errors, and the unidentified cofounders may affect outcomes. We only included admitted patients, so the study data does not include those who died at the scene upon arrival to the ED, those discharged after the ED assessment, or those who died after the discharge. We do not routinely document geriatric-specific assessments like the frailty index or futility, pre-injury location, and geriatric trauma outcome score, and our registry lacks functional results at discharge. Although we lack a DGU, we recently instituted a geriatric service consult for all admitted injured patients above 65 years and for a selected group of younger patients (≥ 60). Moreover, data suggest that the modified FI-5 performs well and aligns with more comprehensive tools like the original FI or its modification, the mFI-11; it is easily obtainable from the TQIP database at admission and is simple to calculate. However, it cannot gauge the severity of each item, which represents an inherent limitation and may account for some of the contradictory findings.

One strength of this first report from the state of Qatar, and, to our knowledge, the most extensive description of the Middle East, is that there is sparse literature on this crucial and growing subpopulation at high risk for severe trauma in the region (Arab Middle East). We have used our findings to develop local clinical protocols to help clinicians take better care of these patients and increase their awareness of how age affects their outcomes and disposition. Furthermore, the recommendations for the ACS-COT [55] and others [56–58] have galvanized us to form a Geriatric Trauma Care Program within our trauma system. There is excellent potential for providing specialized care with close monitoring to impact mortality, functional outcomes, and associated costs of care.

These findings have also provided evidence for targeted injury prevention initiatives that will better address safety at home for the geriatric population of Qatar. The prevention of falls at home that affect the elderly has been prioritized by the Public Health Department of the Ministry of Public Health as it begins the process of creating national injury prevention guidelines. Similarly, the U.S. Department of Health and Human Services (DHHS) launched an initiative (Healthy People 2030) that identified the prevention of falls in the elderly as a national priority to reduce ED visits from this crucial mechanism [59]. In future perspectives, this work could help regionally investigate this vital problem and contribute to understanding its complexity and implications. We also believe that the age cutoff for geriatric trauma should be 55. There is a need to develop quality indicators, study these patients, and generate robust evidence to inform care and prevention. Lastly, females represented only 17.5% of the study population, which could indicate a gender-biased result; however, this is a real reflection of the gender distribution among injured patients in Qatar [60, 61].

## Conclusion

In Qatar, 1 out of 11 trauma admissions were older than 55 years with a male predominance (4:1). Falls at home were the main MOI, with TBIs and bleeding as the two leading causes of mortality in this population. Qatar’s trauma system has created a geriatric trauma service and prioritized injury prevention for this population; however, the age cutoff needs further elaboration to address the growing public health problem of geriatric trauma. The modified FI-5 performs well as a predictive tool in trauma patients with different age groups.

## Data Availability

All data were presented in the manuscript and tables.
